# Stromal expression of miR-21 in T3-4a colorectal cancer is an independent predictor of early tumor relapse

**DOI:** 10.1186/s12876-015-0227-0

**Published:** 2015-01-22

**Authors:** Won Kyung Kang, Jin Kwon Lee, Seong Taek Oh, Sung Hak Lee, Chan Kwon Jung

**Affiliations:** 1 Department of Surgery, College of Medicine, The Catholic University of Korea, Seoul, Republic of Korea; 2 Department of Hospital Pathology, College of Medicine, The Catholic University of Korea, 222 Banpodaero, Seocho-gu, Seoul 137-701 Republic of Korea

**Keywords:** Colorectal neoplasms, Neoplasm recurrence, microRNA, Cadherins, MTA-1 protein

## Abstract

**Background:**

MicroRNA-21 (miR-21) is an oncogenic microRNA that regulates the expression of multiple cancer-related target genes. miR-21 has been associated with progression of some types of cancer. Metastasis-associated protein1 expression and loss of E-cadherin expression are correlated with cancer progression and metastasis in many cancer types. In advanced colorectal cancer, the clinical significance of miR-21 expression remains unclear. We aimed to investigate the impact of miR-21 expression in advanced colorectal cancer and its correlation with target proteins associated with colorectal cancer progression.

**Methods:**

From 2004 to 2007, 277 consecutive patients with T3-4a colorectal cancer treated with R0 surgical resection were included. Patients with neoadjuvant therapy and distant metastasis at presentation were excluded. The expression of miR-21 was investigated by in situ hybridization. Immunohistochemistry was used to detect E-cadherin and metastasis-associated protein1 expression.

**Results:**

High stromal expression of miR-21 was found in 76 of 277 (27.4%) colorectal cancer samples and was correlated with low E-cadherin expression (*P* = 0.019) and high metastasis-associated protein1 expression (*P* = 0.004). T3-4a colorectal cancer patients with high miR-21 expression had significantly shorter recurrence-free survival than those with low miR-21 expression. When analyzing colon and rectal cancer separately, high expression of miR-21 was an independent prognostic factor of unfavorable recurrence-free survival in T3-4a colon cancer patients (*P* = 0.038, HR = 2.45; 95% CI = 1.05-5.72) but not in T3-4a rectal cancer patients. In a sub-classification analysis, high miR-21 expression was associated with shorter recurrence-free survival in the stage II cancer (*P* = 0.001) but not in the stage III subgroup (*P* = 0.267).

**Conclusions:**

Stromal miR-21 expression is related to the expression of E-cadherin and metastasis-associated protein1 in colorectal cancer. Stage II colorectal cancer patients with high levels of miR-21 are at higher risk for tumor recurrence and should be considered for more intensive treatment.

## Background

Colorectal cancer (CRC) is the third most commonly diagnosed cancer in Korea [1]. The prognosis of CRC is associated with tumor progression; five-year survival rates range from 93% to 8% [2]. There are many proposed serological and molecular markers as predictive and prognostic indicators of CRC; however, they are not widely accepted as providing reliable prognostic information due to a lack of reproducibility, validation and standardization among studies [3,4]. Therefore, there is a need to identify more reliable prognostic mediators of tumor progression and metastasis in order to define the behavior of CRC and improve postoperative treatment strategies.

MicroRNAs are small noncoding RNA molecules, 18-25 nucleotides in length, which post-transcriptionally regulate gene expression by binding to the 3’ untranslated regions of target messenger RNAs and play a central role in regulation of mRNA expression [5]. MicroRNAs have been shown to influence all cellular processes [6] and have a high degree of sequence conservation among distantly related organism, indicating their likely participation in essential biological processes [7]. Of note, microRNAs have been reported to have a marked influence on carcinogenesis through the dysregulation of oncogenes and tumor suppressor genes [8]. Cancer-related microRNAs typically show altered expression levels in tumors as compared to the level of expression in the corresponding normal tissue.

MicroRNA-21 (miR-21) is an oncogenic microRNA that regulates the expression of multiple cancer-related target genes, such as *PTEN* and *PDCD4*, and has been reported to be consistently up-regulated in various types of cancers, including colon, breast, lung, and stomach cancers [9-16]. MiR-21 is known to contribute to the regulation of apoptosis, cell proliferation and migration [9,11,17]. Moreover, miR-21 levels increase in the advanced stages of cancer, suggesting a central role for miR-21 in invasion and dissemination of cancer [12,14]. In CRC tissue samples, miR-21 expression is up-regulated during tumor progression and is also known to be associated with poor survival and response to chemotherapy [12,13,18]. However, the clinical significance of miR-21 expression in advanced CRC remains unclear.

In situ hybridization (ISH) for microRNA has an advantage over quantitative microRNA expression analysis platforms in that ISH allows for precise histological localization of microRNAs in formalin-fixed paraffin-embedded tissue blocks [19,20].

Loss of E-cadherin expression is associated with activation of epithelial-mesenchymal transition, invasion and metastasis in various cancers [21]. Conversely, expression of Metastasis-associated protein1 (MTA1) is correlated with cancer progression and metastasis in numerous cancer types, including CRC [22,23]. Previous studies on the association between MTA1 and E-cadherin have shown that MTA1 regulates E-cadherin expression through AKT activation in prostate cancer, and that low E-cadherin expression promotes cancer metastasis [21,24]. However, the exact role of these proteins in CRC remains unclear.

We investigated miR-21 expression using ISH in specimens from T3-4a CRC patients treated by surgical resection. We also evaluated the relationship between expression of miR-21, E-cadherin and MTA1 and their clinical significance as potential biomarkers for prognosis of T3-4a CRC patients.

## Methods

### Patients

From January 2004 until June 2007, a total of 526 consecutive patients underwent surgical resection for CRC at Seoul St. Mary’s Hospital. Of these, 277 patients with pathological T3 (invasion of the subserosa or pericolic/perirectal adipose tissue) or T4a (serosal invasion) cancer were selected for the study, based on the following inclusion criteria: (i) no neoadjuvant chemotherapy or radiation therapy, (ii) no evidence of direct invasion into adjacent structures or organs, (iii) no postoperative death within six weeks, and (iv) no distant metastasis at presentation. The patients consisted of 181 males and 96 females (mean age 63.0 years). Overall survival (OS) was defined as the time interval between surgery and death from any cause or the most recent follow-up date. Recurrence-free survival (RFS) was defined as the time from the date of surgery to the date of first cancer recurrence or the most recent disease-free follow-up. This study was approved by the Institutional Review Board of Seoul, St. Mary’s Hospital, The Catholic University of Korea. Written informed consent was obtained from all patients.

### Tissue microarray construction

We constructed tissue microarrays from formalin-fixed, paraffin-embedded tissues as previously described [25,26]. Two 2-mm-diameter tissue cores were collected from each representative tumor specimen and inserted in a recipient paraffin block. The tissue microarray blocks were serially cut into 4-μm-thick sections for immunohistochemistry and 6-μm-thick sections for ISH.

### Immunohistochemistry for E-cadherin and MTA1

Immunohistochemical staining was performed using specific antibodies against E-cadherin (4A2C7, Zymed, South San Francisco, CA), MTA1 (A-11, Santa Cruz Biotechnology, Santa Cruz, CA) and the Polink-2 plus polymer HRP detection system (Golden Bridge International, Mukilteo, WA, USA) according to each manufacturer’s protocol. The specificity of each antibody was confirmed using both Western blotting and immunocytochemistry in several cell lines with known protein expression status. Negative controls were performed by the substitution of the primary antibodies with normal mouse IgG at the same concentration as the primary antibodies. Multi-tissue blocks containing known-positive tumor tissues were used as positive controls. Staining was examined in triplicate by two gastrointestinal pathologists (CKJ and SHL) who were blinded to the clinicopathological data. Specimens with discordant interpretations were reviewed until an agreement was reached. Immunohistochemical staining results were only assessed by a semiquantitative score of staining intensity (0, no; 1, weak; 2, moderate; 3, strong staining) because nearly all positively-staining tumors showed a diffuse staining pattern for both proteins. These scores were subsequently used to group samples into two categories: low (0 or 1) and high staining (2 or 3). Membrane staining of E-cadherin was evaluated and scored as ‘2’ when tumor cells displayed staining intensity similar to that seen in normal colonic mucosa. MTA1 expression was evaluated as nuclear staining.

### *In situ* hybridization for miR-21

ISH was performed using the miRCURY locked nucleic acid (LNA) microRNA Detection FFPE microRNA ISH Optimization Kit 2 (Exiqon, Vedbaek, Denmark) in a StatSpin ThermoBrite Slide Hybridizer (Fisher Scientific, Westwood, MA) as previously described [19]. We used a double-digoxigenin-labeled LNA miR-21 probe (Exiqon, sequence: 5′-TCAACATCAGT-CTGATAAGCTA-3′), a positive control LNA U6 snRNA probe (Exiqon, sequence: 5′- CACGAATTTGCGTGTCATCCTT-3′) and a negative control LNA scrambled microRNA probe (Exiqon, sequence: 5′- GTGTAACACGTCTATACGCCCA-3′). Tissue sections were counterstained with nuclear fast red. Semiquantitative assessment of the ISH staining results was performed by two pathologists (CKJ and SHL) who were unaware of the clinicopathological and immunohistochemical data. In all cases where disagreements occurred, a consensus was reached by the investigators. The intensity of the staining was scored as negative (0), weak (1), moderate (2), or strong (3), as previously described [27,28], and samples were subsequently grouped into two categories: low (0 or 1) and high (2 or 3) expression.

### Statistical analysis

The relationships between the expression of miR-21, E-cadherin and MTA1 and the clinicopathological parameters were analyzed using the Chi-square test. Cumulative incidence curves for OS and RFS were plotted using the Kaplan–Meier method. The long-rank test was used to detect differences among groups. Multivariate analysis for OS and RFS was conducted using the Cox proportional hazard regression model. All statistical analyses were performed using SPSS, version 16 (SPSS Inc., Chicago, IL). A *p* value <0.05 was considered significant.

### Meta-analysis for the association of miR-21 expression and patient survival

Two authors (CKJ and SHL) performed literature searches using PubMed, Embase databases and Google up to November 2014, and independently selected eligible articles. Inclusion criteria include 1) being related to the association between miR-21 expression and CRC prognosis, 2) original articles, and 3) sufficient RFS or OS data including hazard ratio (HR) with a 95% confidence interval (CI). We performed a meta-analysis of HR of the effect of miR-21expression on RFS or OS in colon or rectal cancer patients. Heterogeneity among studies was assessed using Cochran Q test and *I*^2^ values. A *P* < 0.10 or *I*^2^ > 50% was considered significant heterogeneity. If statistical heterogeneity was observed, the random effect model was used for meta-analysis. Otherwise, we used a fixed-effect model for the meta-analysis. Meta-analyses were performed using Comprehensive Meta Analysis Version 2.0 (Biostat Inc., Englewood, NJ).

## Results

Demographic and clinicopathological variables of the study participants are listed in Table 1.

**Table 1:** **Correlations of clinicopathological parameters and expression of miR-21 in 277 patients with T3-4a colorectal cancer**

Parameter	N	miR-21 expression		*p*-value
		High	Low	
Age				
<65	139	36 (25.9%)	103 (74.1%)	0.565
≥65	138	40 (29.0%)	98 (71.0%)	
Gender				
Male	181	51 (28.2%)	130 (71.8%)	0.705
Female	96	25 (26.0%)	71 (74.0%)	
Primary site				
Right colon	82	20 (24.4%)	62 (75.6%)	0.636
Left colon	91	28 (30.8%)	63 (69.2%)	
Rectum	104	28 (26.9%)	76 (73.1%)	
Histologic type				
Non-mucinous	262	73 (27.9%)	189 (72.1%)	0.767
Mucinous	15	3 (20.0%)	12 (80.0%)	
Differentiation				
Well or moderately	260	74 (28.5%)	186 (71.5%)	0.168
Poorly	17	2 (11.8%)	15 (88.2%)	
Depth of invasion				
pT3	228	65 (28.5%)	163 (71.5%)	0.388
pT4a	49	11 (22.4%)	38 (77.6%)	
Lymph node metastasis				
Absent	138	39 (28.3%)	99 (71.7%)	0.759
Present	139	37 (26.6%)	102 (73.7%)	
AJCC stage				0.118
IIA	122	34 (27.9%)	88 (72.1%)	
IIB	16	5 (31.2%)	11 (68.8%)	
IIIB	82	28 (34.1%)	54 (65.9%)	
IIIC	57	9 (15.8%)	48 (84.2%)	
Perineural invasion				
Absent	219	58 (26.5%)	161 (73.5%)	0.490
Present	58	18 (31.0%)	40 (69.0%)	
Lymphatic invasion				
Absent	120	33 (27.5%)	87 (72.5%)	0.984
Present	157	43 (27.4%)	114 (72.6%)	
Vascular invasion				
Absent	255	72 (28.2%)	183 (71.8%)	0.311
Present	22	4 (18.2%)	18 (81.8%)	
CEA (ng/dL)^a^				
<5	163	46 (28.2%)	117 (71.8%)	0.542
≥5	78	25 (32.1%)	53 (67.9%)	
Adjuvant therapy				
No	16	1 (6.2%)	15 (93.8%)	0.079
Yes	261	75 (28.7%)	186 (71.3%)	
E-cadherin				
Low	109	40 (36.7%)	69 (63.3%)	0.019
High	161	37 (23.0%)	124 (77.0%)	
MTA1				
Low	168	37 (22.0%)	131 (78.0%)	0.004
High	102	39 (38.2%)	63 (61.8%)	

^a^Preoperative serum level of carcinoembryonic antigen (CEA) was measured in 241 colorectal cancer patients. AJCC, American Joint Committee on Cancer. Immunohistochemistry for E-cadherin and MTA1 was available in 270 cases.

### miR-21 expression by in situ hybridization

**Figure 1:**
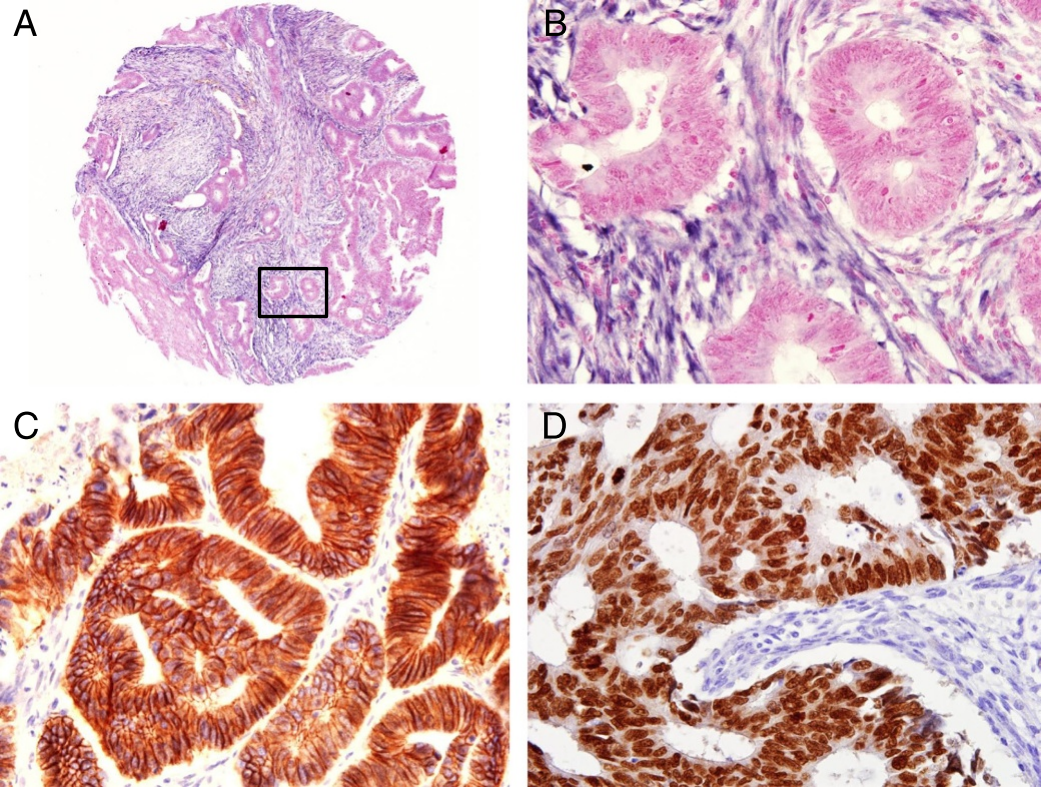
**In situ hybridization for miR-21 and immunohistochemistry for E-cadherin and MTA1. (A)** A representative 2 mm tumor tissue core from the colorectal cancer tissue microarray shows diffuse strong miR-21 expression in the stroma. **(B)** High-magnification image of insert in **(A)** shows that miR-21 signals are strong in the stromal cells of colorectal cancer but not in the tumor cells. Magnification x400. **(C)** Tumor cells show strong membranous expression of E-cadherin. Magnification x400. **(D)** Tumor cells show strong nuclear expression of MTA1. Magnification x400.

miR-21 expression was found to be predominantly localized to the stroma surrounding the tumor cells (Figure 1). High levels of miR-21 were found in 76 of 277 (27.4%) CRC specimens. There was no significant correlation between high miR-21 expression and the clinicopathological features of the patients (Table 1).

### Correlation between miR-21 and MTA1/E-cadherin expression

The expression patterns of E-cadherin and MTA1 in stained tumor cells were membranous and nuclear, respectively (Figure 1). Low expression of E-cadherin was found in 109 of 277 (39.4%) CRCs, and high MTA1 expression was seen in 102 (36.8%) tumors. High miR-21 expression was significantly correlated with low E-cadherin expression (*P* = 0.019) and high MTA1 expression (*P* = 0.004) (Table 1). E-cadherin expression was negatively correlated with MTA1 expression (*P* = 0.005).

### Recurrence-free survival and overall survival

**Figure 2:**
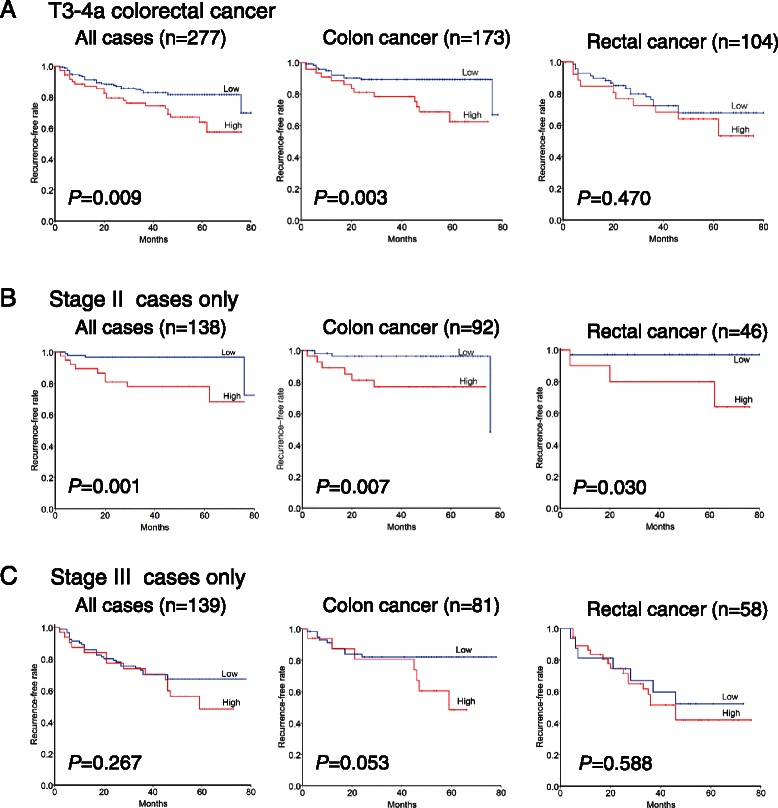
**Association between miR-21 expression and recurrence-free survival in patients with T3-4a colorectal cancer.** Kaplan-Meier survival curves for recurrence-free survival in all **(A)**, stage II **(B)** and stage III **(C)** cancer patients according to miR-21 expression status. **(A)** High miR-21 expression is associated with recurrence-free survival in colon cancer patients but not in rectal cancer patients. **(B)** For the 138 patients with stage II cancer, the association between high miR-21 expression and recurrence-free survival is statistically significant only in colon cancer patients. **(C)** Among 277 stage III cancer patients, high miR-21 expression is not associated with poor recurrence-free survival.

In all 277 CRC patients, variables significantly associated with RFS included miR-21 expression (*P* = 0.010, Figure 2A), histological differentiation (*P* = 0.031), pT stage (*P* = 0.0005), lymph node metastasis (*P* = 0.00001), and serum CEA level (*P* =0.006) (Table 2). In a multivariate analysis, high levels of miR-21 (*P* = 0.007, HR = 2.24; 95% CI = 1.25-4.02), pT stage, lymph node metastasis, and serum CEA level were independent prognostic factors for unfavorable RFS (Table 3). However, the OS rate was not associated with the expression levels of miR-21, E-cadherin or MTA1.

**Table 2:** **Univariate analysis for overall recurrence-free survival among patients with T3-4a colorectal cancer**

	Colorectal cancer (n = 277)		Rectal cancer (n = 104)		Colon cancer (n = 173)	
Variables	HR (95% CI)	*p*-value	HR (95% CI)	*p*-value	HR (95% CI)	*p*-value
miR-21 expression (low vs. high)	2.02 (1.18-3.45)	0.010	1.32 (0.62-2.85)	0.474	3.09 (1.41-6.76)	0.005
Age (<65 years vs. ≥65 years)	0.97 (0.57-1.66)	0.920	0.45 (0.20-1.02)	0.055	2.12 (0.95-4.77)	0.068
Tumor type (non-mucinous vs. mucinous)	1.18 (0.37-3.78)	0.783	3.03 (0.70-13.05)	0.138	0.67 (0.09-4.94)	0.693
Differentiation (well or moderately vs. poorly)	2.56 (1.09-6.00)	0.031	4.57 (1.33-15.67)	0.016	2.06 (0.60-7.00)	0.249
pT (T3 vs. T4a)	2.68 (1.51-4.76)	0.0005	3.97 (1.73-9.12)	0.001	2.30 (1.00-5.30)	0.044
Lymph node metastasis (absent vs. present)	3.69 (1.98-6.87)	0.00001	6.65 (2.30-19.22)	0.0004	2.24 (1.00-5.02)	0.045
CEA (<5 ng/dL vs. ≥5 ng/dL)	2.24 (1.26-3.99)	0.006	2.26 (1.02-5.05)	0.046	2.23 (0.97-5.15)	0.060
Adjuvant therapy (no vs. yes)	4.19 (0.58-30.35)	0.156	NA	NA	3.23 (0.44-23.98)	0.252

HR, hazard ratio; CI, confidence interval; NA, not available.

**Table 3:** **Multivariate analysis of prognostic factors predicting overall recurrence-free survival according to cancer location**

	Colorectal cancer (n = 277)		Rectal cancer (n = 104)		Colon cancer (n = 173)	
Variables	HR (95% CI)	*p*-value	HR (95% CI)	*p*-value	HR (95% CI)	*p*-value
miR-21 expression (low vs. high)	2.24 (1.25-4.02)	0.007	1.65 (0.65-4.16)	0.295	2.45 (1.05-5.72)	0.038
Age (<65 years vs. ≥65 years)	1.03 (0.56-1.89)	0.924	0.27 (0.10-0.70)	0.007	2.48 (1.00-6.12)	0.049
Tumor type (non-mucinous vs. mucinous)	0.61 (0.13-2.97)	0.539	0.62 (0.03-11.50)	0.751	1.03 (0.13-8.48)	0.976
Differentiation (well or moderately vs. poorly)	2.18 (0.83-5.71)	0.114	2.60 (0.55-12.21)	0.225	1.56 (0.41-5.94)	0.513
pT (T3 vs. T4a)	1.97 (1.01-3.83)	0.046	2.26 (0.75-6.79)	0.145	2.27 (0.86-5.97)	0.098
Lymph node metastasis (absent vs. present)	4.55 (2.23-9.29)	0.00003	11.75 (3.33-41.48)	0.0001	3.02 (1.22-7.47)	0.017
CEA (<5 ng/dL vs. ≥5 ng/dL)	2.63 (1.46-4.74)	0.001	3.32 (1.39-7.51)	0.006	2.65 (1.13-6.21)	0.025
Adjuvant therapy (no vs. yes)	2.48 (0.32-19.32)	0.386	NA	NA	2.15 (0.26-18.08)	0.431

HR, hazard ratio; CI, confidence interval; NA, not available. Multivariate analysis is adjusted for age (<65 years vs. ≥65 years), tumor type (non-mucinous vs. mucinous), differentiation (well or moderately vs. poorly), pT (T3 vs. T4a), lymph node metastasis (absent vs. present), CEA (<5 ng/dL vs. ≥5 ng/dL) and adjuvant therapy (no vs. yes).

To further understand the association of prognostic factors and RFS according to the primary cancer site, we analyzed their HRs for RFS in colon and rectal cancer separately (Tables 3 and 4). High expression of miR-21 was associated with shorter RFS in patients with T3-4a colon cancer (n = 173, *P* = 0.005, Figure 2A), but not in patients with T3-4a rectal cancer (n = 104, *P* = 0.474, Figure 2A).

**Table 4:** **Characteristics of studies that evaluated the association between the high expression of miR-21 and recurrence-free survival or overall survival in colorectal cancer**

First author (reference)	Year	Origin	No. of cases	AJCC stage	Recurrence-free survival		Overall survival		Cut-off value	Statistic analysis	Detection method
					HR	95% CI	HR	95% CI			
Schetter [13]	2008	USA	^a^CC 71	I-IV	NA	NA	2.7	1.3-5.5	Third tertile	Multivariate	RT-PCR
		China	^a^CC 103	I-IV	NA	NA	2.4	1.4-4.1	Dichotomize	Multivariate	Microarray
Shibuya [18]	2010	Japan	CRC 156	I-IV	0.396	0.186-0.897	0.513	0.280-0.956	Mean	Multivariate	RT-PCR
Nielsen [19]	2011	Denmark	CC 129	II	1.28	1.06-1.55	1.17	1.02-1.34	Dichotomize	Multivariate	ISH
			RC 67	II	0.85	0.73–1.01	0.97	0.83-1.13	Dichotomize	Multivariate	ISH
Kjaer-Frifeldt [20]	2012	Denmark	CC 764	II	1.41	1.19-1.67	1.05	0.94-1.18	Mean log	Multivariate	ISH
Zhang [29]	2013	China	CC 138	II	1.98	0.95-4.15	NA	NA	Dichotomize	Univariate	RT-PCR
			CC 137	II	1.88	0.95-3.75	NA	NA	Dichotomize	Univariate	RT-PCR
			CC 255	II	1.79	1.22-2.62	NA	NA	Dichotomize	Univariate	RT-PCR
Bovell [30]	2013	USA	CRC 55	IV	NA	NA	3.25	1.37-7.72	Mean	Multivariate	RT-PCR
Toiyama [31]	2013	Japan	CRC 166	I-IV	NA	NA	0.59	0.21-1.63	3.7	Multivariate	RT-PCR
Chen [32]	2013	Taiwan	CRC 195	I-IV	NA	NA	1.655	0.992-2.762	Mean	Univariate	RT-PCR
Hansen [33]	2014	Denmark	CC 554	II	1.348	1.032-1.760	1.075	0.889-1.301	Dichotomize	Multivariate	RT-PCR
Oue [34]	2014	Japan	CC 156	I-IV	NA	NA	1.80	0.91-3.58	Third tertile	Multivariate	RT-PCR
			CC 87	II-III	NA	NA	3.13	1.20-8.17	Third tertile	Multivariate	RT-PCR
		Germany	CC 145	II	NA	NA	2.65	1.06-6.66	Third tertile	Multivariate	RT-PCR
Present study		Korea	CC 173	II-III	3.09	1.41-6.76	0.425	0.142-1.271	Dichotomize	Multivariate	ISH
			RC 104	II-III	1.32	0.62-2.85	2.046	0.557-7.513	Dichotomize	Multivariate	ISH

^a^Only including patients with typical adenocarcinoma. AJCC, American Joint Committee on Cancer; CI, confidence interval; HR, hazard ratio; NA, not available; RT-PCR, reverse-transcription PCR; ISH, in situ hybridization; CC, colon cancer; RC, rectal cancer; CRC, colorectal cancer.

The T3-4a CRC patients were divided into subgroups according to American Joint Committee on Cancer stage*.* In the stage II (T3-4aN0M0) subgroup, we found that patients with high miR-21 expression level had a significantly shorter RFS time than those with low miR-21 level regardless of the primary site (colon cancer, *P* = 0.007; rectal cancer, *P* = 0.030, Figure 2B). However, in the stage III (T3-4aN1M0) subgroup, there was no significant difference in RFS between patients with high or low levels of miR-21 expression (colon cancer, *P* = 0.053; rectal cancer, *P* = 0.588, Figure 2C).

### Meta-analysis

**Figure 3:**
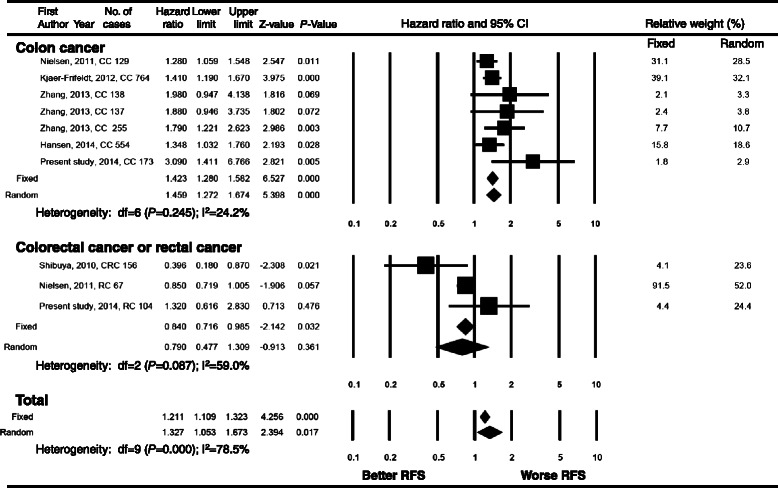
**Forest plot of meta-analysis for the association of high miR-21 expression and recurrence-free survival in colorectal cancer patients.** There is a statistically significant association between high miR-21 expression and poor recurrence-free survival in colon cancer patients. The observed association is not statistically significant in rectal cancer. CI, confidence interval; CC, colon cancer; RC, rectal cancer; CRC, colorectal cancer; RFS, recurrence-free survival.

**Figure 4:**
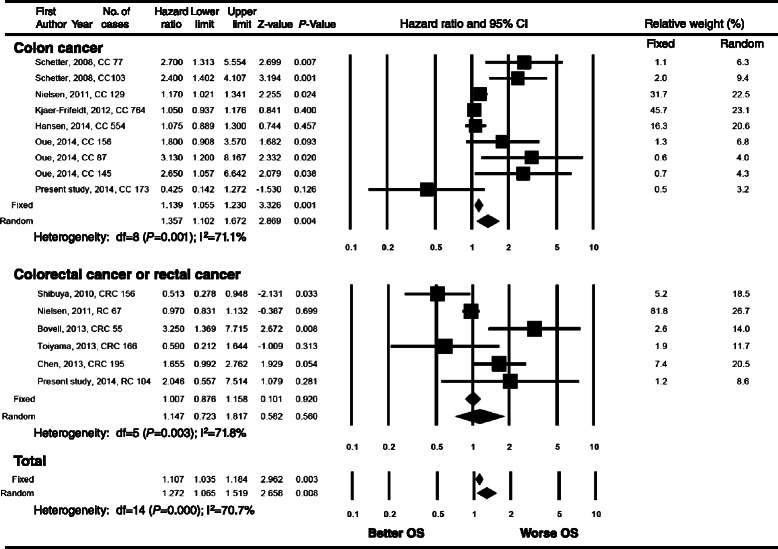
**Forest plot of meta-analysis for the association of high miR-21 expression and overall survival in colorectal cancer patients.** High miR-21 expression is associated with poor overall survival in colon cancer patients but not in rectal cancer. CI, confidence interval; CC, colon cancer; RC, rectal cancer; CRC, colorectal cancer; OS, overall survival.

A total of 10 studies were included for the meta-analysis and their characteristics are summarized in Table 4 [13,18-20,29-34]. High heterogeneity was found in the analysis. For all CRC patients, high miR-21 expression was significantly associated with poor RFS (HR = 1.327, 95% CI = 1.053-1.673, Figure 3) and poor OS (HR = 1.272, 95% CI = 1.065-1.519, Figure 4). In subgroup analysis, the high miR-21 expression was significantly correlated with poor RFS and OS in colon cancer patients (HR = 1.423, 95% CI = 1.280-1.582; HR = 1.357, 95% CI = 1.102-1.672, respectively), but not in rectal cancer or CRC patients.

## Discussion

In the present study, we detected high miR-21 expression in 27.1% (81 of 299) of T3-4a CRCs, and this was associated with low E-cadherin expression and high MTA1 expression. The multivariate analysis revealed that high miR-21 expression was an independent predictor of tumor recurrence in patients with T3-4a CRC.

We observed that miR-21 overexpression occurred in the stroma rather than in the actual tumor cells. Previous studies have reported that miR-21 predominantly localizes to fibroblast-like cells within the tumor-associated stroma of CRC, breast cancer and esophageal cancer [19,35,36]. Using high sensitivity TaqMan quantitative RT-PCR assays in microdissected tissue, Bullock et al. found that miR-21 expression was undetectable in CRC tumor cells but was present in the tumor-associated stroma [35]. Up-regulated miR-21 expression in CRC-associated stroma was associated with transforming growth factor TGF-β-dependent fibroblast-to-myofibroblast transformation and with decreased expression of reversion-inducing cysteine-rich protein with Kazal motifs [35]. The authors proposed that myofibroblast-derived factors mediated tumor progression, and that miR-21 promoted chemo-resistance and tumor invasion by increasing matrix metalloproteinase 2 activity [35]. These results suggest that miR-21 may regulate tumor progression through modulation of the tumor microenvironment.

Recent studies have shown that high stromal miR-21 expression, as measured by ISH, is correlated with shorter RFS in stage II colon cancer [19,20]. In the analysis of OS in stage II colon cancer patients, Nielsen et al. [19] reported on the prognostic significance of miR-21; while Kjaer-Frifeldt et al. [20] were unable to show any significant impact on OS. In the present study using ISH, we found that stromal miR-21 expression was a prognostic factor for RFS in stage II CRC patients but not in stage III patients. Therefore, miR-21 overexpression may have an important role in tumor progression and recurrence prior to the development of lymph node or distant metastases. We found no prognostic value for miR-21 in our analysis of OS, which was calculated as the time from surgery to time of death from any cause.

In the stratified meta-analysis by tumor site, we found that high miR-21 expression was associated with shorter RFS and worse OS in colon cancer, but not in rectal cancer or CRC. The RFS results are consistent with findings of present study.

It has been reported that MTA1 regulates E-cadherin expression via AKT activation in prostate cancer, and that miR-21 is required for regulation of phosphorylated AKT expression in glioblastoma multiforme [24,37]. Xiong et al. suggested that miR-21 influences tumor biology through the PTEN/PI-3 K/Akt pathway in CRC [38]. In our immunohistochemical analysis of E-cadherin and MTA1expression, high MTA1 level was associated with low E-cadherin expression. The expression profiles of these proteins were also significantly correlated with miR-21 expression patterns. Taken together, these results led us to hypothesize that MTA1 may negatively regulate E-cadherin expression via high miR-21 expression in CRC. However, further studies will be needed to determine whether there is a direct role for miR-21 in regulation of MTA1 and E-cadherin expression.

Our study has some limitations including the retrospective, single-institution design and the lack of validation of these results in an independent CRC patient population. Thus, further prospective studies are needed to evaluate the prognostic significance of miR-21 expression.

## Conclusion

miR-21 is overexpressed in the stroma of CRC specimens and has strong associations with the expression of E-cadherin and MTA1. A high level of miR-21 is an independent risk factor predictive of early tumor recurrence in T3-4a colon cancer and stage II CRC. Thus, CRC patients with miR-21 overexpression are at higher risk for tumor recurrence and may benefit from more intensive treatment.

## Abbreviations

CRC: Colorectal cancer
miR-21: MicroRNA-21
ISH: In situ hybridization
MTA1: Metastasis-associated protein1
OS: Overall survival
RFS: Recurrence-free survival
HR: hazard ratio
CI: confidence interval
LNA: locked nucleic acid
